# A Smaller Atlas of Illustrations of Clinical Surgery

**Published:** 1896-06

**Authors:** 


**A Smaller Atlas of Illustrations of Clinical Surgery.** By Jonathan Hutchinson, F.R.S. London: West, Newman & Co. 1895.

This volume consists of one hundred and thirty-six plates, most of them being coloured, all of which have appeared during the last five years in the *Archives of Surgery*, but which are now first published in book-form, accompanied by descriptive letterpress giving an adequate explanation of each plate. Mr. Hutchinson, as is well known, is a firm believer in the value of pictorial illustration as an aid to the acquisition of clinical knowledge, and he has spared no pains in producing some of the most beautiful and life-like plates which have ever been published. A large proportion are of skin diseases, in which the author takes such a keen interest, but many illustrate rare forms of disease, such as microcephalus, meningocele, unusual tumours of the breast, &c.

The great majority of the plates have been prepared from original drawings, executed under the author's own supervision, and we cannot but admire the energy and industry which have enabled him to collect illustrations of such rare and interesting diseases. We trust Mr. Hutchinson will be able to continue his collection, and give us the promised second volume of this valuable atlas.

